# Quantifying spatial dynamics of *Mycobacterium tuberculosis* infection of human macrophages using microfabricated patterns

**DOI:** 10.1016/j.crmeth.2023.100640

**Published:** 2023-11-13

**Authors:** Anca F. Savulescu, Nashied Peton, Delia Oosthuizen, Rudranil Hazra, Robert P. Rousseau, Musa M. Mhlanga, Anna K. Coussens

**Affiliations:** 1 Division of Chemical, Systems, & Synthetic Biology, Institute for Infectious Disease & Molecular Medicine, Faculty of Health Sciences, University of Cape Town, Observatory 7925, South Africa; 2 Centre for Infectious Diseases Research in Africa, Institute of Infectious Disease and Molecular Medicine, University of Cape Town, Observatory 7925, South Africa; 3 Infectious Diseases and Immune Defence Division, Walter and Eliza Hall Institute of Medical Research, Parkville, VIC 3052, Australia; 4 Department of Pathology, University of Cape Town, Observatory 7925, South Africa; 5 Department of Medical Biology, University of Melbourne, Parkville, VIC 3052, Australia; 6 Radboud Institute for Molecular Life Sciences (RIMLS), Radboud University Medical Center, 6525 GA Nijmegen, the Netherlands; 7 Epigenomics & Single Cell Biophysics Group, Department of Cell Biology, FNWI, Radboud University, 6525 GA Nijmegen, the Netherlands; 8 Department of Human Genetics, Radboud University Medical Center, 6525 GA Nijmegen, the Netherlands

**Keywords:** *Mtb*, micropattern, MDM, single cell, host-pathogen interactions, phagocytosis, NHLRC2, MTOC, interferon, cell wall

## Abstract

Macrophages provide a first line of defense against invading pathogens, including the leading cause of bacterial mortality, *Mycobacterium tuberculosis* (*Mtb*). A challenge for quantitative characterization of host-pathogen processes in differentially polarized primary human monocyte-derived macrophages (MDMs) is their heterogeneous morphology. Here, we describe the use of microfabricated patterns that constrain the size and shape of cells, mimicking the physiological spatial confinement cells experience in tissues, to quantitatively characterize interactions during and after phagocytosis at the single-cell level at high resolution. Comparing pro-inflammatory (M1) and anti-inflammatory (M2) MDMs, we find interferon-γ stimulation increases the phagocytic contraction, while contraction and bacterial uptake decrease following silencing of phagocytosis regulator *NHLRC2* or bacterial surface lipid removal. We identify host organelle position alterations within infected MDMs and differences in *Mtb* subcellular localization in line with M1 and M2 cellular polarity. Our approach can be adapted to study other host-pathogen interactions and coupled with downstream automated analytical approaches.

## Highlights

• Microfabricated patterns enable quantitative measurements of *Mtb* MDM interactions
• Interferon-**γ** stimulation increases phagocytic contraction of *Mtb*-infected MDMs
• Removal of bacterial surface lipids attenuates phagocytic contraction and entry
• *Mtb* infection leads to alterations in host organelle positioning

## Motivation

*Mycobacterium tuberculosis* (*Mtb*) manipulates a plethora of macrophage responses to survive within its intracellular niche. Limited knowledge exists regarding the events that occur during phagocytosis and if/how *Mtb* manipulates the spatial organization of its new home. The morphological heterogeneity of differentially polarized primary human monocyte-derived macrophages (hMDMs) presents a hurdle for quantitative analysis of these earliest host-pathogen interactions. This study introduces a micropatterning, image-based approach to reduce this heterogeneity, instilling uniform cell morphology and organelle positioning. This toolbox facilitates high-resolution, image-based, quantitative investigations of previously under-studied early interactions between *Mtb* and differentially derived hMDMs at a single-cell level.

## Graphical abstract

**Figure:**
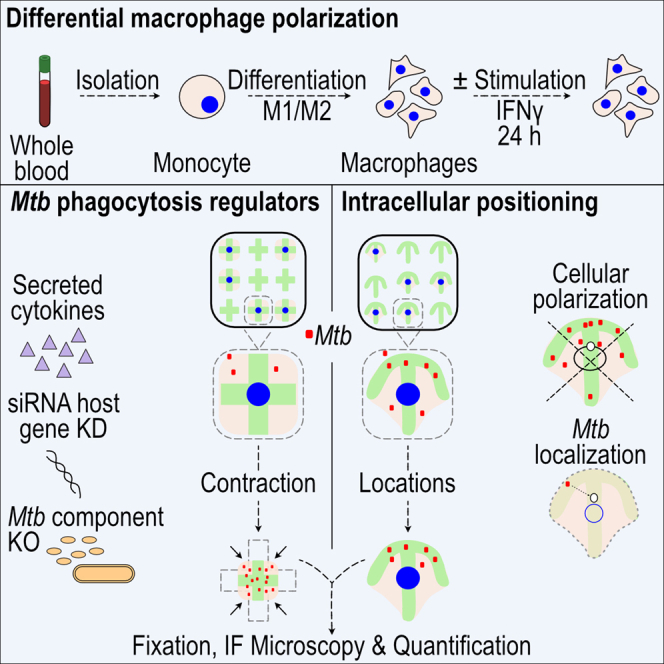

## Teaser

Savulescu et al. present a method quantitatively investigating spatial dynamics of early *Mycobacterium tuberculosis* (*Mtb*) infection. In this approach, primary human macrophages cultured on microfabricated patterns are infected with *Mtb*, allowing phagocytic events and the subcellular position of host organelles and *Mtb* particles to be measured at single-cell, high resolution.

## Introduction

*Mycobacterium tuberculosis* (*Mtb*), the causative agent of tuberculosis disease remains the leading bacterial cause of death world-wide and was responsible for about 1.5 million deaths in 2020.^1^ Macrophages represent an essential cellular component of the innate immune system responding to both acute and chronic inflammatory processes. These immune cells are the first line of defense for the control of pathogens, limiting spread through activation of surface markers, production of pro-inflammatory cytokines and chemokines, and recruitment of peripheral lymphocytes and monocytes to the site of inflammation. With macrophages being the predominant host cells implicated in bacterial entry, growth, and restriction, they have become the immune cell of choice for the study of host-pathogen interactions.^2^ Alveolar macrophages are the primary immune cells to phagocytose inhaled *Mtb* when it reaches the distal airways and thus their functional response is critical to the outcome of infection.^3^

There are two major lineages of macrophage polarization, pro-inflammatory M1 (classically activated) and anti-inflammatory M2 (alternately activated) phenotypes, although additional M2 subphenotypes and other lineages such as M3 and M4 have also been proposed.^4^ Structurally, M1 form a more round cellular phenotype, while M2 are more filamentous and heterogeneous in shape.^5^^,^^6^ Heterogeneity in macrophages induced by mycobacterial infection is marked by altered expression of pro-inflammatory or anti-inflammatory response.^7^ This heterogeneity is particularly relevant when studying complex host-pathogen interactions, when not all cells in a tissue culture dish will be infected, nor undergoing the same process at the same time. Although most studies have routinely used *in-vitro*-generated human monocyte-derived macrophages (MDMs) with great success to analyze cellular responses at a bulk population level, their capacity to differentiate into a homogeneous population remains elusive due to their high degree of plasticity and ability to assume different phenotypes depending on the local microenvironment.^8^^,^^9^^,^^10^ Quantification at the single-cell level mitigates some of the limitations of bulk analyses; however, random dispersal of morphologically heterogeneous cells across a dish still makes systematic quantification of individual cell responses challenging.

To overcome these difficulties, we made use of micropatterning of cells, which imposes morphological homogeneity in cell shape and size, and spatial organization of organelles, without inducing homogeneity in cell function.^11^^,^^12^^,^^13^^,^^14^ This allows us to measure various molecular events and subtle morphological changes quantitatively and do so in a comparative manner between different cell types for events that cannot be quantified in classic 2D cell culture.^13^ Inducing internal consistency of organelle location provides a normalized reference point to start each measurement, while identical starting shape allows deviations from this shape to be quantified. This allows the averaging of a high number of cells and quantitative study of biological processes. We and others have previously demonstrated the advantage that micropatterns convey for quantitative analysis of spatial organization of organelles, including the nucleus, Golgi, and endomembranes,^11^^,^^12^^,^^13^ and RNA transcript measurement and subcellular position,^14^ over 2D cell culture. In addition, others have previously shown during *Salmonella typhimurium* (*Stm*) infection of HeLa epithelial cells that the kinetics of infection are similar using micropatterned cells as they are for conventional 2D culture unconstrained cells; however, the use of micropatterns allows statistical quantification of processes such as *de novo* actin polymerization and rearrangements.^15^ Moreover, as micropatterns are coated in fibronectin coating, or any other matrix substrate of choice, they provide a more physiologically relevant microenvironment for the macrophage cytoskeleton to adhere via integrins compared with a plastic culture dish in which they experience no structural environmental signals.^16^

We made use of this system to study under-characterized early events during and immediately after phagocytosis of *Mtb* by GM-CSF (M1)- and M-CSF (M2)-derived human MDMs at a high resolution, in a quantitative manner, and on a single-cell level. *In vitro* 1D micropatterned adhesive substrates and adhesive microfabricated patterns have been previously used to grow mouse bone marrow-derived macrophages (BMDMs) and peritoneal macrophages,^17^^,^^18^^,^^19^ treated BMDMs and primary peritoneal macrophages grown on micropatterns, with the edema toxin (ET)—the major virulence factor of the anthrax-causing bacterium—*Bacillus anthracis*. ET induced a dynamic disruption of the actin cytoskeleton, eventually leading to cell shrinkage and complete disruption of the cell architecture, including relocalization of the nucleus.^19^ As mentioned above, micropatterns have also been used to analyze F-actin cytoskeleton restructuring induced in HeLa cells by *Stm* infection, demonstrating that docking and invasion sites of *Stm* were preferred to specific cellular height.^15^ In these studies, micropatterning of cells allowed for standardization and quantification of the biological process studied, including cytoskeleton rearrangements and bacterial invasion, as well as statistical cell-to-cell comparison among the cellular population.

Here, we introduce a high-throughput, single-cell, microscopy-based method to study early *Mtb* infection in human MDMs using micropatterned cells. MDMs were grown on standard and elastomeric microfabricated patterns, infected with *Mtb*, and fixed at various time points. Standard patterns allowed quantification of *Mtb* bacilli’s subcellular location relative to organelles, while elastomeric micropatterns allowed quantification of macrophage contraction during *Mtb* phagocytosis. We assessed the impact of an interferon-gamma (IFN-γ) pro-inflammatory microenvironment, blocking a key regulator of actin polymerization, and removing or blocking bacterial cell wall and effector protein secretion on phagocytic contraction and bacterial uptake. We found that *Mtb* infection altered microtubule-organizing center (MTOC) positioning differently in M1 and M2 MDMs and affected the cell nucleus position in both MDM types. A correlation was observed between *Mtb* bacilli’s subcellular location and MTOC positioning, indicative of cellular polarity.

Our method enables visualization and quantification of these processes due to the homogeneous cell populations on micropatterns perturbed by *Mtb* infection. This approach can be applied to high-throughput studies of other pathogen infections in MDMs, facilitating quantitative analyses of host-pathogen interactions.

## Results

### *Mtb* H37Rv infection of MDMs on micropatterns

**Figure 1:**
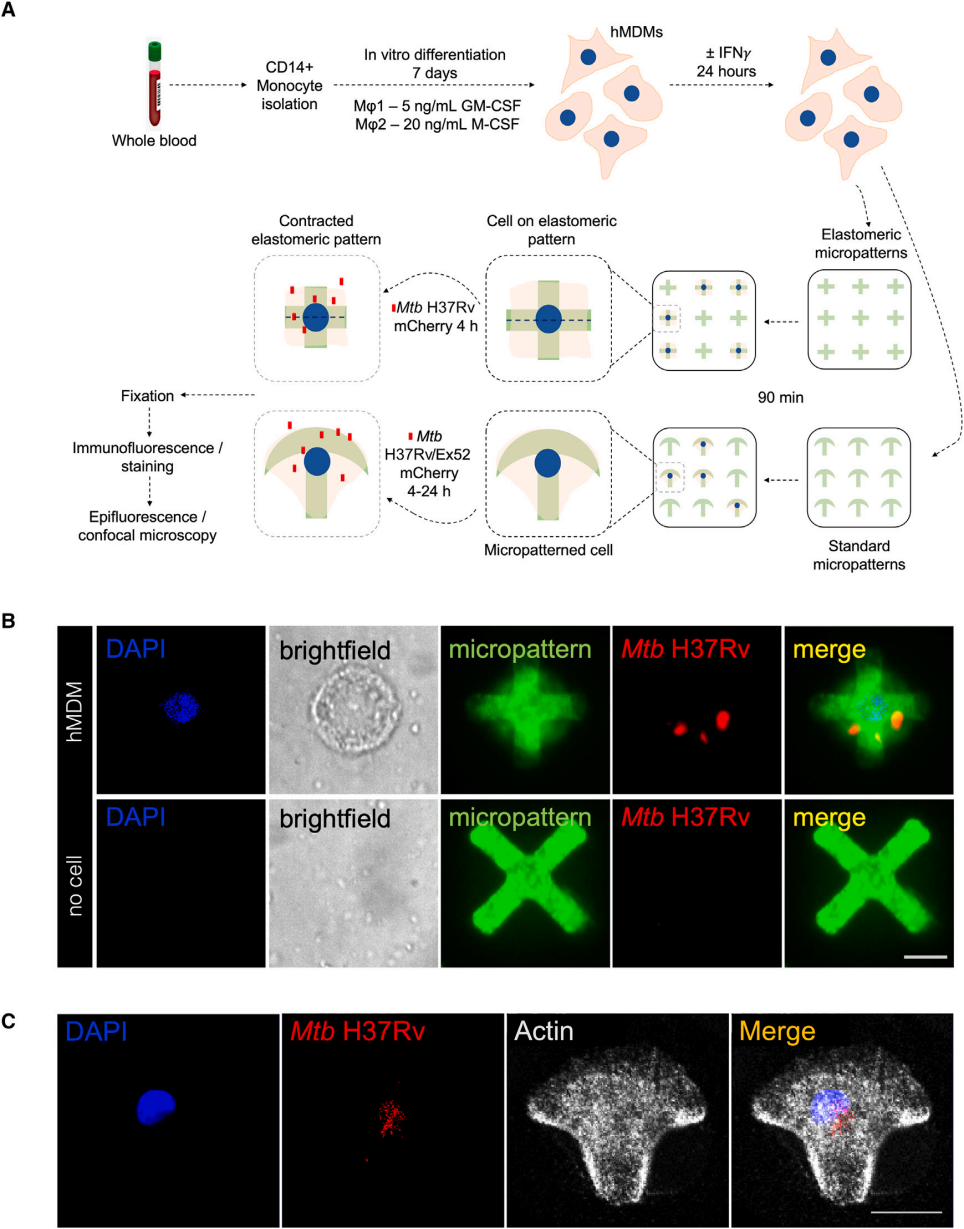
Methodology schematic for *Mtb* infection of human MDMs using elastomeric and standard micropatterns (A) PBMCs are isolated from whole blood of healthy donors by gradient separation followed by CD14^+^ monocyte isolation from PBMCs using magnetic bead positive selection. CD14^+^ monocytes are differentiated into M1 and M2 macrophages followed by overnight stimulation with or without 100 IU/mL IFN-γ. Cells are then seeded at a concentration of ∼75,000 cells per 0.5 mL onto elastomeric or standard patterns, left to adhere and adopt the appropriate shape for 60–90 min, with unbound cells washed prior to infection. Cells are infected with either *Mtb* laboratory strain H37Rv or clinical strain Ex52 for 4 h, and cells washed and either fixed immediately or cultured for up to 24 h. Cells are then fixed for 16–24 h overnight, washed with 1× PBS, and taken for immunofluorescence, followed by imaging using epifluorescence/confocal microscopy. (B) Images of a *Mtb* H37Rv-infected M1 MDM on an elastomeric micropattern (top) and an elastomeric pattern that does not contain a cell (bottom). Contraction of the elastomeric pattern is clearly seen in the top but not in the bottom image. DNA is stained with DAPI (blue), the cell is shown in bright field (gray), the micropattern is labeled with Alexa Fluor 488 (green) and *Mtb* H37Rv expresses mCherry (red). A merged image is shown on the right. Scale bar, 10 μm. (C) An image of a *Mtb* H37Rv-infected M1 MDM on a standard crossbow micropattern. DNA is stained with DAPI (blue), *Mtb* H37Rv expresses mCherry (red), and actin is stained with phalloidin (gray). A merged image is shown on the right. Scale bar, 10 μm.

We first set out to establish a micropatterned-based assay to reduce variation in cell-to-cell structural and organelle spatial organization that would allow quantitative study of early stages of *Mtb* infection in MDMs. To do so, we isolated CD14^+^ monocytes from healthy donor PBMCs and then differentiated them *in vitro* for 7 days in the presence of either GM-CSF or M-CSF to generate M1 and M2 MDM, respectively (Figure 1A). At the site of *Mtb* infection, macrophages are exposed to a cytokine-enriched microenvironment, with IFN-γ induced by *Mtb* effector proteins in infected macrophages and produced by *Mtb*-specific T cells, a hallmark of TB inflammation.^20^ MDM were therefore cultured in the presence or absence of IFN-γ before infection. We then made use of the FLECS (fluorescently labeled elastomeric contractible surfaces) technology^21^ to measure phagocytosis of *Mtb* in MDMs (Figure 1A). In this technique, cells are grown on elastomeric micropatterns and mechanical forces, such as mechanotransduction, phagocytosis, and others, are measured as contractions in the elastomeric micropatterns and quantified at a single-cell level. We seeded MDMs on fluorescently labeled elastomeric micropatterns at a concentration of 75,000 cells/0.5 mL per well in 24-well plates. The cells were left to adhere for 60–90 min, followed by removal of non-adhesive cells by gentle washing. The macrophages were then infected with mCherry fluorescently labeled *Mtb* at a multiplicity of infection (MOI) of 10 for 4 h. This was followed by fixation of cells overnight at 37°C with 4% pre-warmed paraformaldehyde (PFA), 1× PBS washes, and storage at 4°C. Once fixed, we performed immunofluorescence to label actin and DNA, and imaged the cells on a StellarVision microscope, using synthetic aperture optics (SAO). The use of SAO permits low-objective lenses to be used, capturing hundreds of cells, and then, offline, the imaging of individual cells at high resolution. Thus, the length of each individual elastomeric pattern could be accurately measured using image analysis tools. Importantly, only single cells that spread out appropriately on the micropattern were quantified.

In parallel, we prepared micropatterns based on the protocol described in Azioune et al.^22^ In brief, we coated coverslips with poly(L-lysine) poly(ethylene glycol) and made use of a chromium photomask to imprint the desired size and shape of micropatterns onto coated 10 mm coverslips. We then coated the imprinted micropatterns with fibronectin, followed by seeding of MDMs at a concentration of 75,000 cells/0.5 mL per coverslip in 24-well plates. Micropattern sizes ranging from small to large were tested, as well as two different shapes (crossbow and round) (Figure S1). However, medium-sized crossbow-shaped micropatterns were our preferred pattern of choice as they have been shown to promote a polar intracellular organization of cells.^12^^,^^13^ In addition, their size is typical to that of MDMs. The cells were left to adhere for 60–90 min, followed by removal of non-adhesive cells by gentle washing. Both cell types adhered well to the micropatterns, but we found that pre-treatment with 100 IU/mL IFN-γ for 24 h before seeding improved cellular spreading and adoption of the correct micropatterned shape for both cell types. Following removal of the non-adherent cells, macrophages were infected with mCherry/GFP fluorescently labeled *Mtb* at a MOI of 10 for 4 h. This was followed by fixation of cells at 4, 16, or 24 h post infection using 4% pre-warmed PFA, 1× PBS washes, and storage at 4°C. We then performed immunofluorescence and epifluorescent or confocal microscopy on fixed cells to visualize *Mtb* bacilli and cellular landmarks, including the MTOC and actin structure. This was followed by manual image analysis using the Fiji program to characterize the subcellular localization of *Mtb* bacilli in relation to cellular markers. Only cells that adopted the micropattern shape correctly were quantified. We note that the imaging data can be coupled with automated analytical approaches to quantify subcellular localization patterns and colocalization levels, as in Savulescu et al.^14^ Images of typical MDMs on elastomeric micropatterns and standard crossbow micropatterns are shown in Figures 1B and 1C.

### Elastomeric micropatterns provide a quantitative assay to measure *Mtb* H37Rv phagocytosis in MDMs

**Figure 2:**
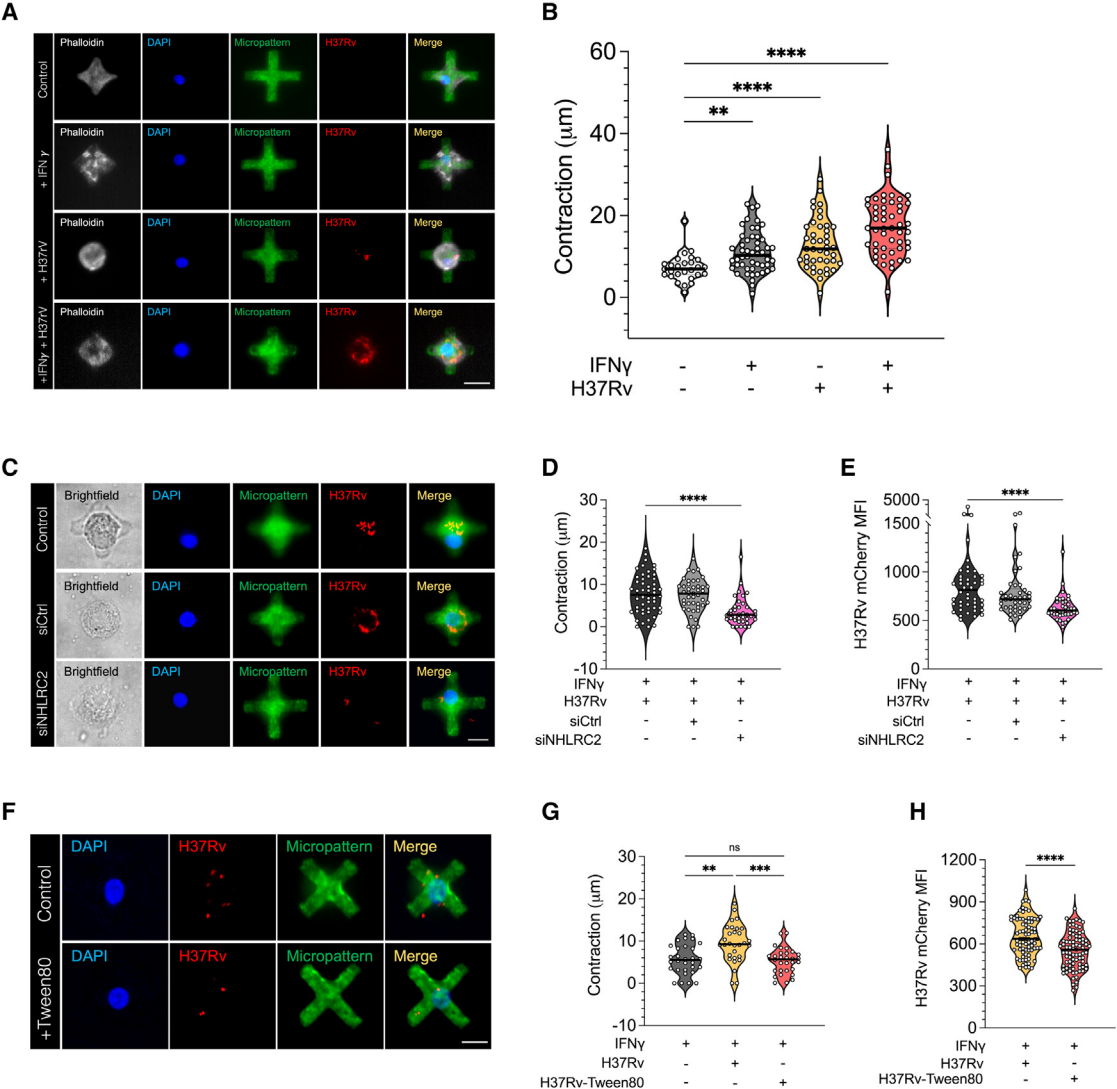
Elastomeric patterns provide a quantitative assay to measure phagocytosis of *Mtb* H37Rv by M1 MDMs (A) Images of MDMs unstimulated/stimulated with IFN-γ and uninfected/infected with H37Rv. Actin is stained with phalloidin (gray), the DNA is labeled with DAPI (blue), the elastomeric pattern is labeled with Alexa Fluor 488 (green), and *Mtb* bacilli express mCherry (red). A merged image is shown on the right. n = 25–50 cells. Scale bar, 25 μm. (B) Contraction (measured in μm) of elastomeric micropatterns in MDMs unstimulated/stimulated with IFN-γ and uninfected/infected with H37Rv. (C) Images of MDM cells infected with H37Rv (Control), MDM cells infected with H37Rv and transfected with non-specific scramble siRNA (siCtrl), and MDM cells infected with H37Rv and transfected with siNHLRC2 (siNHLRC2). A bright-field image is shown on the left (gray), the DNA is labeled with DAPI (blue), *Mtb* bacilli express mCherry (red) and the elastomeric pattern is labeled with Alexa Fluor 488 (green). A merged image is shown on the right. Scale bar, 20 μm. (D) Contraction of elastomeric micropatterns in infected MDMs, treated with IFN-γ, that were either transfected with non-specific scramble siRNA (siCtrl) or siNHLRC2, or untransfected, n = 3 donors. (E) Mean fluorescence intensity (MFI) of H37Rv in MDM cells treated with IFN-γ that were either transfected with siCtrl or siNHLRC2, or untransfected, n = 3 donors. (F) Images of MDMs infected with H37Rv (top) and MDMs infected with H37Rv-Tween 80 (bottom). The DNA is labeled with DAPI (blue), *Mtb* bacilli express mCherry (red) and the elastomeric pattern is labeled with Alexa Fluor 488 (green). A merged image is shown on the right. Scale bar, 20 μm. (G) Contraction of elastomeric micropatterns in uninfected MDMs, MDMs infected with H37Rv, and MDMs infected with H37Rv-Tween 80, n = 2 donors. (H) MFI of H37Rv or *Mtb* H37Rv-Tween 80 in MDM cells, n = 2 donors. Violin plot line, median, dotted lines, IQR; analyzed by Kruskall-Wallis test with Dunn’s multiple comparison (B, D, E, G) or Mann-Whitney test (H); ∗∗p < 0.01, ∗∗∗p < 0.001, ∗∗∗∗p < 0.0001; ns, not significant.

We made use of the FLECS technology to specifically look at the earliest stage in *Mtb* infection, namely phagocytosis. The elastomeric patterns are cross shaped, in a 24-well plate format, coated with fibronectin and Fibrinogen-Alexa Fluor 488 fluorescent dye. Upon exertion of a mechanical force on cells growing on these micropatterns, the cells and, subsequently, the elastomeric micropatterns, contract. We seeded MDMs on elastomeric patterns, allowed them to adhere and adopt the typical shape for 90 min, washed off unadhered cells, and infected them with *Mtb* H37Rv (a common lineage 4 laboratory strain of *Mtb)* expressing mCherry, for 4 h, followed by fixation. We then stained fixed samples with DAPI and imaged the cells on a StellarVision microscope. The length of the elastomeric patterns was measured manually, using FiJi. Initially, we set out to test whether phagocytosis of H37Rv can be visualized and quantified using these elastomeric patterns. We compared IFN-γ-stimulated cells with unstimulated cells, as well as H37Rv-infected cells with uninfected cells and combinations of these conditions (Figures 2A and 2B). Unstimulated cells that were infected with H37Rv showed a higher level of contraction compared with unstimulated and uninfected cells (p < 0.0001), suggesting that the assay is able to capture phagocytosis. Interestingly, stimulation of cells with IFN-γ in the absence of infection also resulted in contraction compared with untreated cells, although less significant (p = 0.0057). As such, the largest contraction was observed in cells that were both stimulated with IFN-γ and infected with H37Rv (p < 0.0001). This suggests that at *in vivo* sites of disease an IFN-γ microenvironment may increase macrophage *Mtb* phagocytosis.

We next sought to validate the ability of our assay to be used for functional assessment of host and pathogen-mediated mechanisms governing phagocytosis. To do so, we first used siRNA to deplete expression of *NHLRC2* (siNHLRC2), the top hit in a genome-wide CRISPR screen identified as a key regulator of phagocytosis in the human monocytic cell line U937.^23^ We tested the impact of *NHLRC2* silencing on contraction of IFN-γ-treated H37Rv-infected MDMs, the condition with the greatest contraction. siNHLRC2 cells showed a significant decrease in contraction (p < 0.0001) compared with both cells transfected with non-specific scramble siRNA (siCtrl) and untransfected controls; there was no significant difference between siCtrl and untransfected controls (Figures 2C and 2D). To confirm that the contraction is indeed a measurement of phagocytosis, we measured the *Mtb* mCherry mean fluorescence intensity (MFI) in siNHLRC2, siCtrl, and untransfected cells. In line with the level of contraction, a significantly lower mCherry MFI was observed in siNHLRC2 cells compared with siCtrl and untransfected cells (Figure 2E). Taken together, these data demonstrate the functional validity of our assay to study macrophage mechanism of phagocytosis and confirm NHLRC’s role as a regulator of *Mtb* phagocytosis by primary human macrophages.

As it has been shown that *Mtb* strain variation in cell wall surface lipids influences receptor-mediated uptake by macrophages,^24^ next we used the elastomeric patterns to test whether stripping lipids off the surface of *Mtb* H37Rv modulates macrophage contraction during phagocytosis. To do so, we cultured H37Rv mCherry in the absence (H37Rv) or presence of Tween 80 (H37Rv-Tween 80), a detergent commonly used to remove the surface lipids from *Mtb* during broth culture to prevent bacilli from clumping. Single-cell suspension stocks for infection were then prepared in an identical manner using glass beads to dissociate bacterial clumps and create a single-cell suspension with sequential low-speed centrifugation. Bacterial counts were confirmed by CFU plating and then stocks used to infect MDM at an MOI of 10:1 for 4 h. Infecting IFN-γ-stimulated M1 MDMs on elastomeric micropatterns with the two preparations of *Mtb*, consistent with Figure 2A, we again saw a significant increase in contraction when infecting with H37Rv grown in the absence of Tween 80 compared with IFN-γ stimulation only (p = 0.0016). However, this H37Rv-induced contraction was significantly reduced when infecting with H37Rv-Tween 80 (p = 0.0004) to a level of contraction similar to IFN-γ stimulation only (Figures 2F and 2G). To confirm that the significant difference in contraction observed between the two bacterial preparations is indeed a measurement of phagocytosis, we measured the mCherry MFI. In line with the level of contraction, a higher MFI was observed in cells infected with cell wall lipid intact H37Rv compared with cells infected with H37Rv-Tween 80 (p = 0.040) (Figure 2H).

Finally, as *Mtb* ESX1 secretion system effector proteins are known to inhibit macrophage phagosome maturation and induce IFN-γ production,^25^ we tested the effect of infection with dead versus live *Mtb* to assess the impact of blocking effector protein secretion on *Mtb* phagocytosis. To avoid any difference in cell wall composition that may exist between divergent *Mtb* mutant strains that are missing the RD1 locus, which encodes the ESX1 system, we instead chose to take an infection stock of mCherry *Mtb* prepared in the absence of Tween 80, split it in half, and heat killed one aliquot at 85°C for 10 min. We optimized this minimum length of killing to ensure fluorescent bacteria remained intact (Figure S2A)*.* Testing the effect in M1 and M2, we first found that M1 had significantly greater contraction and intracellular *Mtb* MFI using live and dead *Mtb* compared with M2 (Figures S2B and S2C), in line with their pro-inflammatory phenotype. After 4 h infection, M2 had significantly greater contraction when using live versus dead *Mtb* and a similar trend was seen with M1. M1 also showed greater uptake of live versus dead *Mtb*, measured by *Mtb* MFI, with a more subtle effect in M2, given their lower overall phagocytosis of *Mtb* (Figure S2C). Together with our findings using Tween 80, these data confirm the utility of our assay to investigate bacterial cell wall and secreted factors, which modify *Mtb* phagocytosis in a quantitative manner, at a single-cell level.

### Micropatterns are suitable for the study of early but not late stages of *Mtb* H37Rv infection in M1 and M2 MDMs

**Figure 3:**
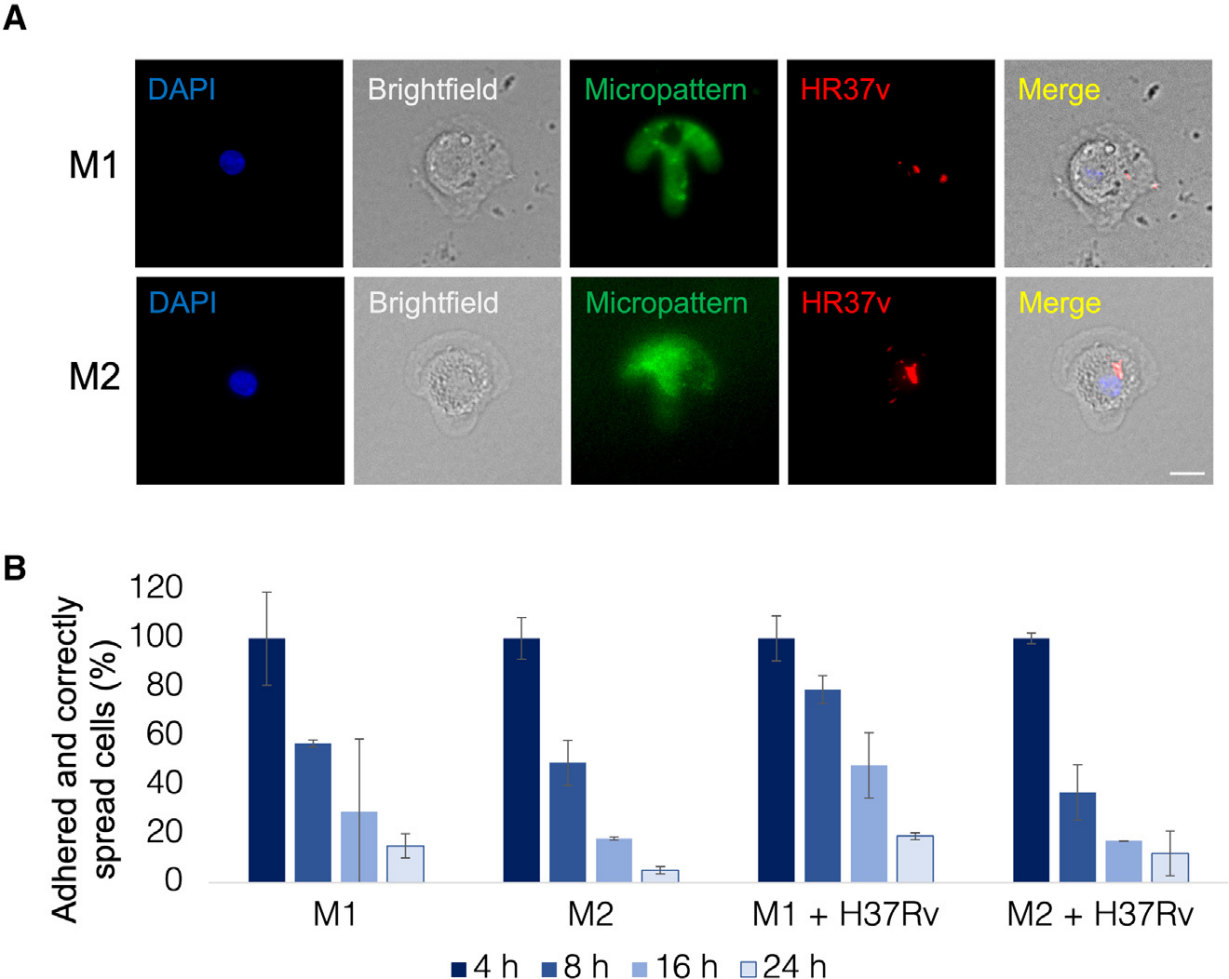
Micropatterns are suitable for early but not late stages of H37Rv infection in MDMs (A) Typical H37Rv-infected M1 and M2 MDMs on crossbow-shaped micropatterns. The DNA is labeled with DAPI (blue), the cell can be seen in bright field, the micropatterns are labeled with Alexa Fluor 488 (green), *Mtb* H37Rv expresses mCherry (red), and a merged image (combining all fields other than the green micropattern, for clarity) is shown on the right. Scale bar, 10 μm. (B) Percentages of appropriately adhered and spread, uninfected, and H37Rv-infected M1 and M2 MDMs on crossbow micropatterns at 4, 8, 16, and 24 h post infection. The number of cells that are adhered and spread in an appropriate manner per condition at 4 h is considered as the 100%, with the following time points calculated as a percentage of the 4 h. n = 3 donors, mean ± SD.

Having used elastomeric patterns to quantify processes involved in *Mtb* phagocytosis, we next made use of standard micropatterns to quantify various intracellular processes following *Mtb* uptake. First, we set out to characterize the time frame over which we could successfully capture micropatterned MDMs following infection with *Mtb* H37Rv. Typically, studies using micropatterned cells explore processes that occur within a short time frame from seeding (2–8 h).^12^^,^^13^^,^^15^^,^^16^^,^^26^^,^^27^^,^^28^^,^^29^^,^^30^^,^^31^ We therefore wanted to establish this time frame for MDMs infected with *Mtb*. We seeded M1 and M2 MDMs on crossbow micropatterns and infected them with mCherry/GFP fluorescently labeled H37Rv for various time points ranging from 4 to 24 h, before fixing. We then stained cells with DAPI and imaged them on a StellarVision microscope at a low magnification to allow the visualization and quantification of hundreds of cells. We manually counted the number of cells on each coverslip and compared the numbers at different time points. The typical number of MDMs that was adhered and spread appropriately on micropatterns was ∼200–250 cells per 10 mm coverslip (Figure S3). For comparison, we seeded M1s and M2s on both crossbow micropatterns without infecting them to test whether infection with *Mtb* H37Rv modifies adherence on micropatterns. Typical images of H37Rv-infected M1 and M2 cells on crossbow-shaped micropatterns are shown in Figure 3A. The DNA is stained with DAPI, the cell is seen in bright field, the micropatterns are labeled with Alexa Fluor 488, and we used an H37Rv fluorescent reporter strain that constitutively expressed mCherry. We set the number of cells that were adhered and appropriately spread on micropatterns at the earliest time point (4 h) as the 100% and calculated the number of cells at each following time point as a percentage of this 4 h time point. Absolute number of adhered and appropriately spread cells on crossbow micropatterns are shown in Figure S3. No significant differences were observed in the number of adhered and appropriately spread cells at 4 h between uninfected M1s and M2s and between infected M1s and M2s (p = 0.832 and 0.476, respectively) (Figure S3). Similarly, no significant differences were observed in adherence and spreading at 4 h between uninfected versus infected M1s or M2s (p = 0.725 and 0.772, respectively) (Figure S3). For both M1s and M2s, a declining trend can be seen from 4 h throughout the timeline to a low of ∼5%–15% cells remaining at the 24 h time point, irrespective of H37Rv infection (Figure 3B). Notably, less uninfected M1s remained adhered to micropatterns compared with infected cells; only ∼55%–60% of uninfected M1s remained adhered to micropatterns at 8 h compared with 70%–80% for infected M1s. Similarly, a slightly higher number of H37Rv-infected M1s remained adhered at the 16 and 24 h time points compared with uninfected M1s (Figure 3B). This suggests that infection on M1s with H37Rv may help adhere or/and stabilize these cells on micropatterns. Curiously, the opposite can be seen for uninfected versus H37Rv-infected M2s at the 8 h time point—infection with *Mtb* H37Rv reduced the number of adhered cells on micropatterns (Figure 3B). However, this reduction is not consistent at later time points (16 and 24 h) (Figure 3B). When translating the percentages into cell numbers, the typical number of adhered and properly spread uninfected and H37Rv-infected M1 and M2 MDMs at 8 h was ∼120–130 (Figure S3), which we considered as an appropriate number of cells that allowed quantification and statistical analyses. However, the number of cells that remained adhered on micropatterns at the 16 and 24 h time points was lower than 75 cells in most cases, which we considered to be insufficient for quantitative and statistical downstream analyses, as previously shown.^13^^,^^15^^,^^32^

In parallel to crossbow-shaped micropatterns, we also made use of round shape micropatterns to test whether the adherence and spreading rate depends on the shape of the micropatterns (Figure S4). Similar trends in all parameters were observed with round-shaped micropatterns (Figure S4). Thus, we conclude that adherence does not depend on the shape of the micropattern when analyzing events up to 24 h post infection in M1 or M2 macrophages. To summarize, we consider the H37Rv infection of MDMs on micropatterns assay to be best suitable for the study of early stages of H37Rv infection up to at least 8 h to provide statistically robust measurements.

### Nucleus and MTOC positionings are altered in *Mtb* H37Rv-infected M1 and M2 MDMs

**Figure 4:**
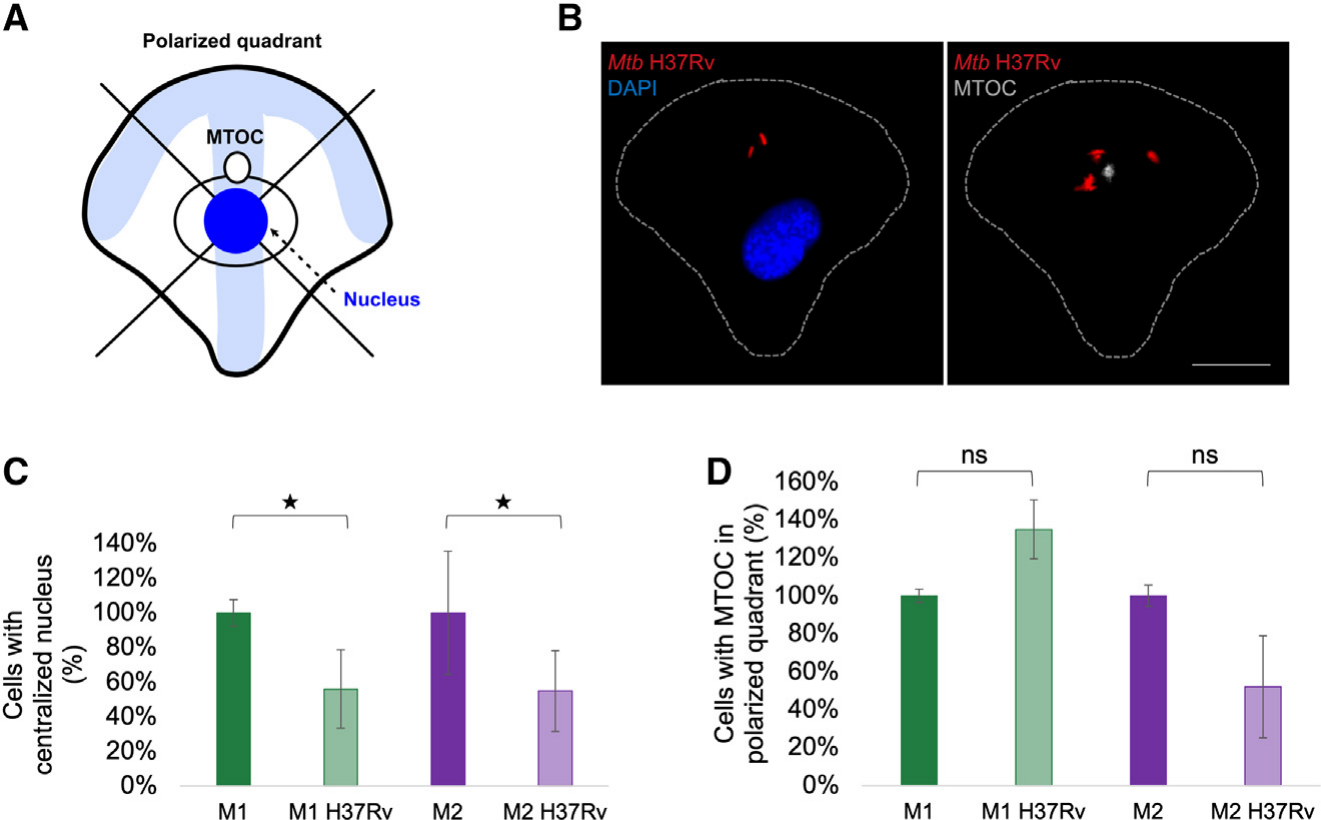
Nucleus and MTOC positionings are altered in H37Rv-infected M1 and M2 MDMs (A) Schematic of a micropatterned cell with typical positions of the nucleus in blue and MTOC in white. Two artificial lines are drawn through the center of the cell to divide it into four quadrants. The top quadrant represents the polarized edge of the cell. (B) Typical images of M1 MDMs on crossbow-shaped micropatterns displaying correct nucleus and MTOC positions. DNA is labeled with DAPI (blue), the MTOC is marked in gray (microtubules and MTOC were stained with an anti-tubulin antibody and the background microtubule staining was subtracted in Fiji to emphasize the MTOC), the contour of the cell is shown in a dotted line, and *Mtb* H37Rv expresses mCherry (red). Scale bar, 10 μm. (C) The percentage of H37Rv-infected MDMs relative to uninfected MDMs (set as 100%), which display centralized location of the nucleus. (D) The percentage of H37Rv-infected MDMs relative to uninfected MDMs (set as 100%), which display MTOC location in the polarized (top) quadrant. Mean ± SD, n = 3 donors for M1 and n = 2 for M2. Analyzed by two-tailed t test (C and D); ∗p < 0.05; ns, not significant.

One of the advantages of using micropatterns is that the 3D localization and position of organelles in adhered cells are maintained in a stable manner, allowing reliable cell-to-cell comparison of organelle spatial relationships and their perturbance by a stimulus. In crossbow-shaped micropatterned cells the nucleus is typically positioned in the center of the cell (Figure 4A).^32^ When artificially dividing the cell into four quadrants as shown in Figure 4A, the MTOC is typically found in the top polarized quadrant, creating an invisible axis from the nucleus, through the MTOC, and toward the leading edge of the cell on the top of the crossbow. We sought to establish whether *Mtb* infection perturbs the typical nucleus and MTOC positioning, comparing H37Rv-infected M1 and M2 MDMs with their respective uninfected control cells. To do so, we seeded M1 and M2 MDMs on crossbow-shaped micropatterns, infected them with H37Rv, and fixed 4 h post infection. We performed immunofluorescence with an anti-tubulin antibody to visualize the microtubules and MTOC, labeled the DNA with DAPI, and imaged the cells on a Zeiss LS880 airyscan confocal microscope, Zeiss Axio Observer 7, or StellarVision microscope. Typically, 60%–70% uninfected M1s and 65%–75% uninfected M2 in a sample display centralized positioning of the nucleus. We set the number of uninfected M1 and M2 MDM cells that displayed a centralized location of their nuclei as 100% and compared the number of H37Rv-infected M1 and M2 MDM cells to that 100%, respectively.

For both M1 and M2 H37Rv-infected MDMs there was a notable reduction in the number of cells that displayed a typical, centralized position of the nucleus (56% and 55% for M1 and M2 MDMs, respectively, displayed centralized nuclear localization; M1 p = 0.029, M2 p = 0.023) (Figure 4C). In cells in which the nucleus was not found in the center, it was typically found off-center toward one of the right, left, or top quadrants (see examples in Figure S5B). Surprisingly, in M1 there was a 35% increase in the number of cells with the MTOC in the polarized quadrant when infected with H37Rv, relative to uninfected, while for M2 a 48% decrease in cells with the MTOC in the polarized quadrant was observed when H37Rv-infected M2 were compared with uninfected M2 (Figure 4D), with a trend toward significance compared with uninfected cells for M1 but not M2 (M1 p = 0.079, M2 p = 0.494). This suggests that, even in H37Rv-infected M1 cells in which the nucleus is not centered, the MTOC is more likely to be located in the correct quadrant compared with infected M2. Taken together, these data suggest that infection with H37Rv might lead to alterations in positioning of cellular organelles both in M1 and M2 MDMs. Example images of centered nucleus and MTOC in H37Rv M1 and M2 MDMs are shown in Figures 4B, and S5A, respectively.

### Laboratory strain H37Rv and *Mtb* clinical isolate Ex52 localize in accordance with the MTOC and cellular polarity

**Figure 5:**
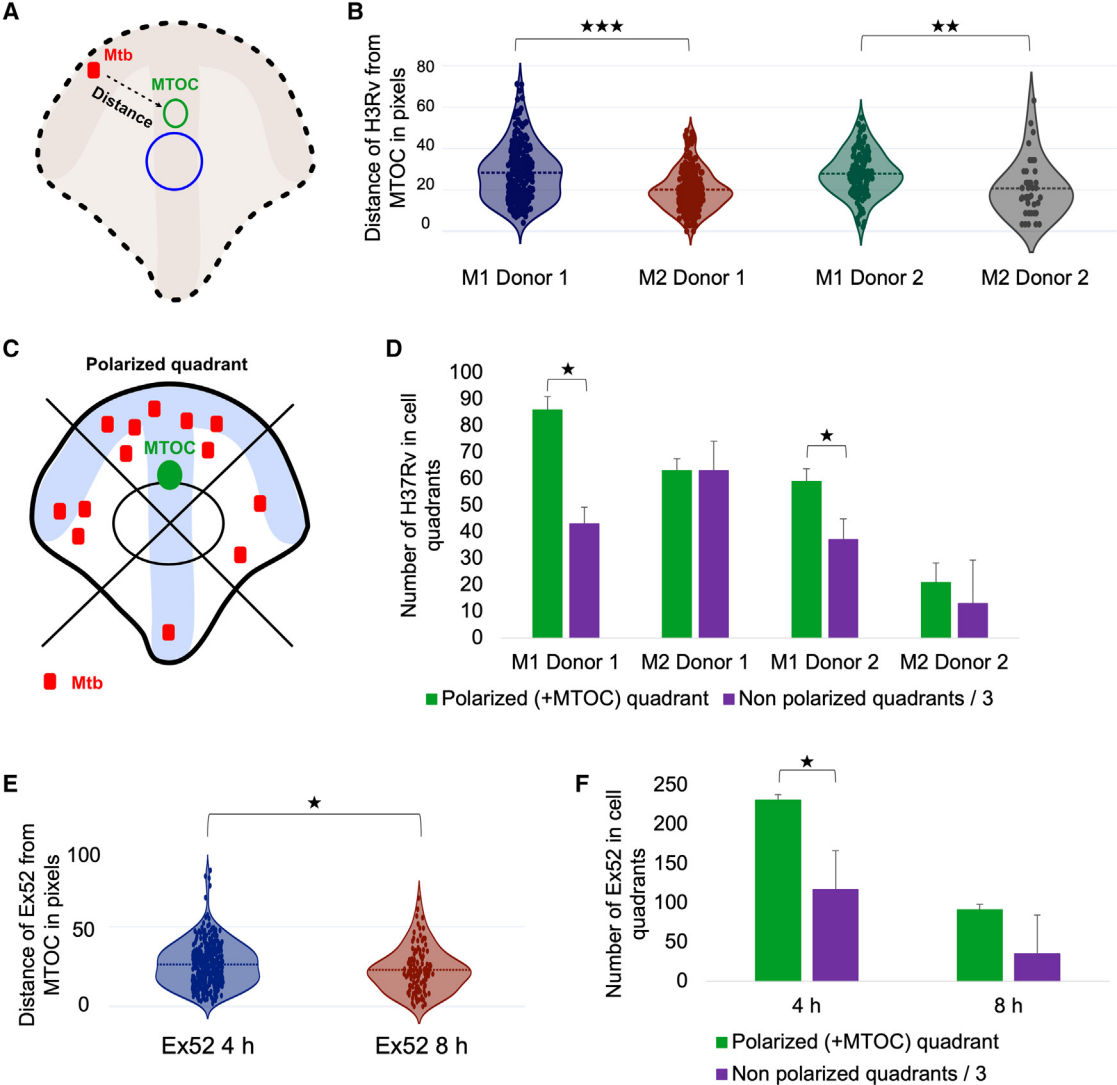
*Mtb* lineage 4 H37Rv and clinical isolate Ex52 localize in accordance to the MTOC and cellular polarity (A) Schematic describing the measurement of distance between each *Mtb* bacillus and the MTOC in pixels. (B) Distance between H37Rv bacilli and the MTOC in pixels for two donors in M1 and M2 MDMs. (C) Schematic describing distribution of *Mtb* bacilli in the different cellular quadrants. *Mtb* bacilli are counted in each quadrant and the average number of *Mtb* bacilli in a non-polarized quadrant is obtained by dividing the sum of all *Mtb* bacilli in the three non-polarized bacilli by three. (D) The total number of H37Rv bacilli in the polarized quadrant compared with the average number of H37Rv in a non-polarized quadrant for M1 and M2 MDMs from two donors. (E) Distance between the clinical strain Ex52 bacilli and the MTOC in pixels in M1 MDMs at 4 and 8 h post infection. (F) The total number of Ex52 bacilli in the polarized quadrant compared with the average total number of Ex52 in a non-polarized quadrant for M1 MDMs at 4 and 8 h post infection. Violin plot line, median; bars plot mean with SD. Analyzed by two-tailed t test; ∗p < 0.05, ∗∗p < 0.01, ∗∗∗p < 0.001.

Finally, we were interested to see whether, once internalized, the *Mtb* bacilli were randomly distributed in space, or positioned in specific locations, which may be in line with the cellular polarity, measured by the nucleus-MTOC-cell edge axis. To do so, we infected crossbow micropatterned M1 and M2 MDMs from two donors with H37Rv and fixed samples 4 h post infection. We then performed immunofluorescence with an anti-tubulin antibody to visualize microtubules and the MTOC, labeled the DNA with DAPI, and imaged cells on an epifluorescence microscope. We manually measured the distance in pixels between each H37Rv bacillus and the MTOC, as described in the schematic in Figure 5A, with 40–225 H37Rv bacilli measured per condition (Figure 5B). Across two donors, the average distance of an H37Rv bacillus from the MTOC was significantly lower in M2s compared with M1s (mean distance 20.1 versus 28.3 pixels, respectively, in the first donor, p = 3.9191 ×10^−12^, and 20.8 versus 27.8 pixels, respectively, in the second donor, p = 0.0053) (Figure 5B). The consistency in distance difference of bacilli from MTOC between M1 and M2 suggests that there may be a functional consequence for the *Mtb* location relative to other organelles depending on cellular phenotype. Further experiments will need to be conducted to characterize the mechanistic insights of this phenomenon.

Next, we counted the number of H37Rv bacilli in each quadrant (Figure 5C) to assess whether there is enrichment of H37Rv in the polarized quadrant. To do so, we divided the number of H37Rv bacilli in all three non-polarized quadrants by three to account for the cell volume in each quadrant (assuming that the volumes of the four quadrants are roughly equal)^14^ and compared this relative number with the number of *Mtb* H37Rv in the polarized quadrant. For M1 MDMs in both donors, a 2-fold enrichment of H37Rv bacilli was found in the total number of bacilli in the polarized quadrant compared with the total number of bacilli in non-polarized quadrants (p = 0.046 and 0.020 for donor 1 M1 and donor 2 M1, respectively) (Figure 5D). Conversely, there was no *Mtb* enrichment in the polarized quadrant observed in M2 for either donor (p = 0.207 and 0.216 for donor 1 M2 and donor 2 M2, respectively) (Figure 5D). Donor 2 M2 also had the lowest uptake of *Mtb* of all cells tested (21 versus 13 H37Rv in the polarized versus non-polarized quadrants). To summarize, these data suggest that coordinated spatial positioning of H37Rv relative to the MTOC and cell polarity differs between M1 and M2 MDM, as well as emphasizing donor variation that is inherent to human primary cell cultures and can be systematically quantified at subcellular levels using micropatterns.

Our data so far were compiled with the most commonly used lineage 4 laboratory strain *Mtb* H37Rv. We next sought to determine if the localization of *Mtb* bacilli in relation to the MTOC and cell polarity can also be observed, and remain consistent, with a lineage 4 clinical isolate of *Mtb*. For this, we seeded M1 MDMs on crossbow-shaped micropatterns, infected them with clinical *Mtb* Ex52, which was transformed with a constitutive mCherry fluorescent reporter, and fixed them 4 or 8 h post infection, to observe localization of Ex52 in relation to the MTOC and cell polarity over time. As for H37Rv, we measured the distance of each Ex52 bacillus from the MTOC, as well as counting the number of Ex52 in each quadrant. Interestingly, the mean distance of Ex52 bacilli from the MTOC was lower at 8 versus 4 h post infection (20.8 versus 25.8 pixels, respectively, p = 0.0229) (Figure 5E), suggesting a potential tighter relative positioning with the MTOC and cell polarity over time. Finally, we found the total number of Ex52 bacilli in the polarized quadrants to be almost 2-fold enriched compared with the non-polarized quadrants (231 versus 117 bacilli, respectively at 4 h, p = 0.016, and 91 versus 35 bacilli, respectively, at 8 h, p = 0.072), consistent with our observation with H37Rv in M1 at 4 h (Figure 5F). Taken together, these data confirm that, for both the laboratory strain H37Rv and the clinical isolate Ex52, there appears to be a correlation with the MTOC positioning and cell polarity in M1 MDM.

## Discussion

Here, we introduce a single-cell image-based approach to visualize and quantify host and pathogen factors that regulate *Mtb* phagocytosis by primary human macrophages and the spatiotemporal location of *Mtb* and host organelles in differentially polarized macrophages, at a high resolution, in a high-throughput manner. Our method is based on micropatterning of MDMs to reduce cell-to-cell variation in morphology and intracellular organelle location, allowing for direct comparison of heterogeneous single cells. The use of micropatterns to study bacterial infection has been explored in the past, treating BMDMs with the ET originating from *Bacillus anthracis*^19^ and infecting the HeLa cell line with *Stm*.^15^ However, to the best of our knowledge, this is the first time that bacterial infection using clinical and laboratory isolates is shown in micropatterned primary human macrophages. Furthermore, we are able to measure the mechanotransductive force in phagocytosis of *Mtb* undergoing phagocytosis by primary human macrophages.

We first sought to use an elastomeric micropattern assay to characterize a very early stage in *Mtb* infection of macrophages, namely phagocytosis. More specifically, we quantified the level of micropattern contraction as a measure of H37Rv phagocytosis. Indeed, cells infected with H37Rv displayed contraction compared with uninfected cells, confirming that the elastomeric patterns assay can indeed be used to study phagocytosis of *Mtb* in MDMs. Due to the necessity to fix and sterilize cells prior to performing imaging measurements to quantify contractive forces during *Mtb* phagocytosis outside of the pathogen containment laboratory, it is possible there is a small unquantified impact of fixation on contractive force measurements. However, previous studies have demonstrated the utility of PFA fixation to measure very subtle changes in contractile forces in other cell types.^21^ Furthermore, we showed that pre-treatment with IFN-γ increased contraction during *Mtb* infection, while, conversely, decreasing expression of *NHLRC2*, removing surface lipids from *Mtb* by culturing in the presence of Tween 80, or inhibiting effector protein secretion by killing the bacteria we can inhibit phagocytic contraction, and reduce bacterial uptake, measured by subcellular *Mtb* fluorescence. These findings demonstrate a functional use for this assay to study the involvement of microenvironment modulation and host and pathogen factors in phagocytic processes on a single-cell level and in a quantitative manner.

We then turned to standard micropatterns to study various aspects in *Mtb* infection. First, we optimized the assay to find the best suitable micropattern shape and size for MDM cells in general, and their performance for *Mtb*-infected MDMs. We then characterized the time frame for infection using our assay. Our data showed that early but not late stages of H37Rv infection can be studied in M1 and M2 MDMs, as cells start to detach from micropatterns at later time points. This detachment after 8 h is consistent with previous studies using micropatterns to study early biological processes 2–8 h after an event.^12^^,^^13^^,^^15^^,^^16^^,^^26^^,^^27^^,^^28^^,^^29^^,^^30^^,^^31^^,^^32^ We did not see increased detachment with *Mtb* infection; in fact, infection with H37Rv seemed to increase the adherence of M1 but not M2 MDMs on micropatterns. This observation is in line with previous studies that showed that *Mtb* infection increases cellular adhesion and surface presentation of adhesion molecules β2 integrin LFA-1 (CD11a) and its counter receptor ICAM-1 (CD54) in primary human macrophages^33^^,^^34^ and that M1 have higher expression of CD54, which also contributes to their rounder shape compared with elongated M2.^5^ We would like to note that other types of micropatterns can be used to assess later events, such as positioning of organelles, cell polarity, alignment of podosomes, recapitulation of embryo regionalized cell fate, and more.^35^^,^^36^^,^^37^^,^^38^

As we propose our assay for the quantitative study of spatial subcellular localization of host-pathogen biomolecules and factors during initial stages of infection, during and immediately following phagocytosis, we set out to explore whether infection of MDMs with the *Mtb* laboratory strain H37Rv affects the spatial positioning of key organelles. We specifically chose the nucleus of the cell, as it adopts typical positioning in micropatterned cells, as well as the MTOC, as it indicates the general cellular polarity. Interestingly, we observed opposite effects in the MTOC cellular positioning in M1 and M2 MDMs upon infection with H37Rv, namely an increase in the number of M1s that display typical MTOC positioning and a decrease in the number of M2s displaying typical positioning of the MTOC. These initial observations lay the foundation for future research to gain mechanistic insights into these potential differences and what impacts donor variability. One possible explanation could be the different level of heterogeneity between M1s and M2s. More specifically, as M2s are more heterogeneous in shape, they would be expected to display more differences within a donor and between donors, depending on the level of differentiation of the cell. Typical centered location of the nucleus was observed in both M1 and M2 MDMs; however, in both cases, infection with H37Rv reduced the number of cells with centralized nuclei. This may suggest the potential restructure of organelles or/and the cytoskeleton that might occur as a result of H37Rv infection, which can be investigated in future studies. Our findings demonstrate the utility of microfabricated patterns to investigate such phenomena and quantify the interaction between *Mtb* and organelle spatial organization.

In addition to studying the effects of H37Rv infection on organelle positioning, we also looked at the subcellular localization of H37Rv bacilli particularly in relation to the MTOC, which is indicative of cellular polarity. Interestingly, we found that the mean distance of H37Rv from the MTOC in M2 MDMs is shorter compared with M1 MDMs. We also found that there is an enrichment of H37Rv in the polarized quadrant of M1 MDMs compared with the non-polarized quadrants, but this was not consistently observed in M2 MDMs. Similar results for M1 were obtained with the clinical strain *Mtb* Ex52. Taken together, these data suggest that *Mtb* localizes internally in correlation to the cell polarity. It remains to be seen whether this preference for localization within the polarized cell space extends to its extracellular interactions, leading to preferred internalization of *Mtb* via the polarized edge, or whether the spatial organization occurs internally via a yet unknown mechanism. Imaging at earlier time points will address this question. Interestingly, although H37Rv is located further away from the MTOC in M1 MDMs compared with M2 MDMs, there is still a distinct enrichment of *Mtb* H37Rv in the polarized edge of the cell. A possible explanation would be that the early phagosome aligns with the nucleus-MTOC-leading edge axis and the general cellular polarization. Tran et al.,^39^ offer another perspective in polarized epithelial cells infected with *Pseudomonas aeruginosa*. Bacterial aggregates on the apical membrane created a localized NF-κB host response and change in host spatiotemporal polarity, acting as an immune-activating signal. M1 MDMs may use a similar approach where *Mtb* is more enriched in the polarized quadrant to aid immune cell activation. Future research can gain further insights of this correlation between *Mtb* location and cellular polarity.

Taken together, by reducing cell-to-cell variability, we were able to compare between cells in a population and quantify subcellular localization patterns of *Mtb* in relation to the MTOC, as well as alterations in host organelle position post *Mtb* infection, and differences between individuals. These data would have been challenging to collect and quantify in standard cell culture, given the heterogeneity of the cell population. This is particularly the case with subtle changes in subcellular localizations of host and pathogen biomolecules or organelles, induced by *Mtb* infection. Furthermore, variability in qualitative and quantitative observations in host-pathogen interactions, which occur between blood donors used for deriving primary macrophages, are more readily assessed using micropatterning. The variability stems from variation in the genetic and epigenetic background of donors, as well as differences associated with donor gender and age, which might affect host-pathogen interactions. Our protocol is particularly suited to study this variability in host-pathogen interactions among the population, as by controlling the experimental system and reducing cell to cell variability, including cell size, shape, morphology, and spatiotemporal localization of organelles, variability in host-pathogen interactions can be reduced to specific differences between donors.

To summarize, our assay is single-cell based and is suitable to study early stages of *Mtb* infection, during and immediately following phagocytosis, in MDMs in a quantitative and high-throughput manner. Although our analysis was done manually, using Fiji, our assay can readily be coupled with automated analytical approaches, such as the one described in Savulescu et al.^14^ These analytical pipelines should be able to uncover patterns of subcellular distribution of biomolecules and organelles and enable quantitative, in-depth characterization of spatial distribution patterns. This assay can be used to study the spatial distribution of a variety of pathogen or host-derived biomolecules at early stages of *Mtb* infection of MDMs, in a quantitative manner, at the single-cell level. Furthermore, we propose that this assay can be modified to study additional pathogenic infections in MDMs, or, alternatively, optimized to study *Mtb* infection in additional cell types.

### Limitations of the study

The limitations of this study include the following: (1) the subcellular localization of an *Mtb* particle is defined in relation to a chosen cellular marker, in our case, the MTOC, or nucleus, and is not an absolute parameter; (2) the annotation of the nucleus and MTOC has been done manually, automation is difficult in this system, as the signal labeling tubulin antibody may have a low signal-to-noise ratio, and the annotation often requires manually looking through a few Z-slices (we expect the annotation to be automated shortly); (3) however, while human MDMs spread well on elastomeric and standard micropatterns, not all cell lines and primary cells spread as well (for example, HEK293T, THP-1s, etc.), which limits the assay to adherent cell types; (4) direct comparisons between morphological features of micropatterned cells and 2D culture cannot be conducted, as in 2D culture there is no consistent reference point that can be used to anchor specific measurements; (5) it is possible that in rare instances there is a small unquantified effect of fixation of cells on contractile force measurements in elastomeric patterns. Notably, fixation is a prerequisite for the intracellular staining technique and imaging described in our study; (6) cells on micropatterns do not come in contact with neighbor cells, and hence cellular processes affected or influenced by cell-cell contact cannot be studied.

## STAR★Methods

### Key resources table

**Table:** 

REAGENT or RESOURCE	SOURCE	IDENTIFIER
**Antibodies**		

Anti-Tubulin	Abcam	Cat# ab6160; RRID:AB_305328
Donkey anti Rat Alexa Fluor 647	Abcam	Cat# ab150151

**Bacterial and virus strains**		

Mycobacterium tuberculosis lineage 4 laboratory strain	Abrahams et al., 2012^40^	H37Rv-GFP
*Mycobacterium tuberculosis* lineage 4 laboratory strain	Center for Infectious Disease Research in Africa	H37Rv-pCherry3
*Mycobacterium tuberculosis* lineage 4 clinical isolate	Center for Infectious Disease Research in Africa	Ex52-pCherry3

**Biological samples**		

Human Adult Blood	Western Province Blood Transfusion Service, Cape Town, South Africa.	HREC #317/2016

**Chemicals, peptides, and recombinant proteins**		

Fibronectin	Thermo Fisher Scientific (Gibco)	Cat# 3010018
DAPI	Life Technologies	Cat# D1306
Vectashield	LSBio	Cat# LS-J1032
PLL(20)-g[3.5]- PEG(2)	Surface Solutions (SuSoS)	N/A
Formaldehyde	SigmaAldrich	Cat# 50-00-0
0.1% Triton X-100	SigmaAldrich	Cat# 9036-19-5
Phalloidin–Atto 565	SigmaAldrich	Cat# 94072
Difco™ Middlebrook 7H9 broth	Becton Dickinson	Cat# 271310
Middlebrook ADC enrichment	Becton Dickinson	Cat# 211887
Tween 80	Sigma-Aldrich	Cat# P1754
Dulbecco’s phosphate-buffered saline	Sigma-Aldrich	Cat# D8537
Glycerol	Sigma-Aldrich	Cat# G5516
Difco™ Middlebrook 7H10 agar	Becton Dickinson	Cat# 262710
Lymphoprep™	Alere Technologies	Cat# 1114547
RPMI 1640 media	Sigma-Aldrich	Cat# R8758
L-glutamine	Sigma-Aldrich	Cat# G8540
Sodium pyruvate	Sigma-Aldrich	Cat# S8636
Human AB serum	Sigma-Aldrich	Cat# H3667
Human GM-CSF	Miltenyi Biotec	Cat# 130-093-864
Human M-CSF	Miltenyi Biotec	Cat# 130-096-485
Recombinant Human Interferon gamma	R&D Systems	Cat# 285-IF-100
Accutase	Sigma-Aldrich	Cat# A6964
TRIzol reagent	Life Technologies	Cat# 15596018
Chloroform – isoamyl alcohol mixture	Sigma-Aldrich	Cat# 25666
2-Propanol	Sigma-Aldrich	Cat# I9516
Sodium acetate	Sigma-Aldrich	Cat# 71196
Linear Acrylamide	Life Technologies	Cat# AM9520
Ethyl Alcohol	Sigma-Aldrich	Cat# E7023
0.5M EDTA	Sigma-Aldrich	Cat# E03690
Tris-EDTA	Sigma-Aldrich	Cat# E93283
Water	Sigma-Aldrich	Cat# W3513

**Critical commercial assays**		

CD14 MicroBeads, human	Miltenyi Biotec	Cat# 130-118-906
MACS™ Miltenyi LS columns	Miltenyi Biotec	Cat# 130-042-401
High Capacity cDNA synthesis kit	Applied Biosystems	Cat# 4368814
Fast SYBR™ Green Master Mix	Applied Biosystems	Cat# 4385612
Mirus TransIT-X2 Dynamic Transfection System	Mirus Bio LLC	Cat# MIR6004

**Deposited data**		

Raw microscopy data	lead contact, available upon request	N/A

**Oligonucleotides**		

Primer: *RPL13A* forward: GAAAAAGCGGATGGTGGTTC	Shireen et al., 2022^41^	N/A
Primer: *RPL13A* reverse: CCGGTAGTGGATCTTGGCT	Shireen et al., 2022^41^	N/A
Primer: *NHLRC2* forward: CGGAAGGATTAGAATGGCTG	This paper	N/A
Primer: *NHLRC2* reverse: GCAGCAGTAGGTGAAGAAATCAAG	This paper	N/A

**Recombinant DNA**		

pCherry Vector	Tan et al., 2013^42^	
NHLRC2 Silencer Select siRNAs	Thermo Fisher (Ambion)	Cat# s51529, s51530, s51531
Scramble Silencer Select siRNA control	Thermo Fisher (Ambion)	Cat# 4390843

**Software and algorithms**		

FiJi/ImageJ (2.0.0)	Schindelin et al., 2012^43^	N/A
GraphPad Prism-8, Version 8.0	GraphPad Prism (https://graphpad.com)	N/A

**Other**		

Glass beads 2 mm	Sigma-Aldrich	Cat# Z273627
MaxTract High Density tubes	Qiagen	Cat# 129056
Eppendorf Safe Lock 2mL tubes	Qiagen	Cat# 0030120.094
Falcon 15 mL conical centrifuge tubes	ABDOS Life Science	Cat# P10402
Falcon 50 mL conical centrifuge tubes	ABDOS Life Science	Cat# P10404
TPP 6-well TC plates polystyrene	Sigma-Aldrich	Cat# Z707759
TPP 96-well TC plates polystyrene	Sigma-Aldrich	Cat# Z707910

### Resource availability

#### Lead contact

Further information and requests for resources should be directed to and will be fulfilled by the Lead Contact, Anna K. Coussens, coussens.a@wehi.edu.au.

#### Materials availability

This study did not generate new unique reagents.

#### Data and code availability

• The data analyzed in this study, including raw microscopy data or any additional information required to analyze the data is available from the lead contact upon request.
• No code was generated in this study.
• Any additional information required to analyze the data reported in this paper is available from the lead contact upon request.

### Experimental model and study participant details

#### Human blood samples

Acquisition of human blood samples and the experiments that were performed with them were approved by the Human Research Ethics Committee of the University of Cape Town (HREC #317/2016). Buffy coats were obtained from anonymous blood donors through the Western Province Blood Transfusion Service, Cape Town, South Africa. Buffy coats were primarily selected from healthy donors aged between 20 and 40 years regardless of gender, ethnicity and socio-economic status. Where possible, gender was equally selected when buffy coats were in abundance.

### Method details

#### Generation of fluorescently-labeled *Mtb* and preparation of *Mtb* infection stocks

500 ng of pCherry3 vector (*smyc*'mCherry; kind gift from David Russell^42^ was electroporated into both H37Rv and clinical Ex52 and plated on 7H10/OADC (BD Biosciences) agar plates containing 50 μg/mL hygromycin. A single colony containing the fluorescently-labeled pCherry3 vector was inoculated into 10 mL 7H9/ADC (BD Biosciences)/0.05% Tween 80 (Sigma) media containing 50 μg/mL hygromycin and grown in static culture at 37°C for 10 days. Cultures were frozen in aliquots in the presence of glycerol at −80°C prior to the preparation of single cell stocks for H37Rv and clinical Ex52. A stock of H37Rv-GFP (a kind gift from the Molecular Mycobacteriology Research Unit, University of Cape Town, South Africa) was inoculated into 10 mL 7H9/ADC (BD Biosciences)/0.05% Tween 80 (Sigma) media containing 25 μg/mL kanamycin and grown in static culture at 37°C for 10 days. Cultures were also frozen in aliquots in the presence of glycerol at −80°C prior to the preparation of single cell stocks.

For single cell stock preparation, frozen parent glycerol stocks were inoculated in 7H9/ADC (BD Biosciences)/0.05% Tween 80 (Sigma) media containing either 50 μg/mL hygromycin (H37Rv-pCherry3 and Ex52-pCherry3) or 25 μg/mL kanamycin (H37Rv-GFP) and grown in static culture at 37°C for 10 days then sub-cultured 1/100 in the same medium in the presence of Tween80 (to generate H37Rv-Tween80) or absence of Tween 80 (to generate H37Rv and Ex52) for a further 10 days. Cultures were pelleted at 3500 x *g* for 10 min, supernatant removed, and pellets vigorously shaken with 2–3 mm glass beads for 1 min. PBS was added to dispersed *Mtb*, which was gently pelleted @ 1400 x *g* for 10 min and the top half of the supernatant, containing single cell *Mtb*, was resuspended with a final volume of 5% glycerol and frozen at −80°C. Before and after freezing, stocks were titrated and plated on 7H10/OADC (BD Biosciences) agar plates containing either 50 μg/mL hygromycin (H37Rv-pCherry3 and Ex52-pCherry3) or 25 μg/mL kanamycin (H37Rv-GFP) for CFU determination. To make heat-killed *Mtb*, 20μL aliquots of *Mtb* stocks were incubated at 85°C in a metal bead bath for increasing 10 min intervals from 10 to 60 min. Following incubation, heat-killed aliquots were transferred into a 96-well plate and *Mtb* mCherry fluorescence captured on a ZOE Fluorescent Cell Imager (Bio-Rad). Following this, 180 μL of 7H9/ADC broth was added to each well and incubated for up to 5 days at 37°C with a 10th volume of alamarBlue cell viability reagent (Thermo Fisher) added and incubated overnight before bacterial growth was fluorescently measured at peak excitation of 570 and emission of 585 nm and color change photographed.

#### Preparation of MDMs

Peripheral Blood Mononuclear cells (PBMCs) were obtained from healthy donor buffy coats, and were isolated using a Lymphoprep (Alere Technologies) density gradient. CD14 monocytes were isolated from PBMCs through CD14^+^ magnetic bead separation (MACS Miltenyi) and differentiated at 4x10^6^ cells per 40 mm dish for 7 days at 37°C in RPMI media (Sigma) supplemented with 1 mM Sodium Pyruvate (Sigma), 2 mM L-Glutamine (Sigma), 10% Human AB Serum (hAB) (Sigma) and 5 ng/mL GM-CSF (MACS Miltenyi) or 100 ng/mL M-CSF (MACS Miltenyi), to generate the macrophage phenotypes M1 and M2, respectively. Following the 7-day differentiation, spent media was removed from plates and replenished with fresh prewarmed RPMI media (Sigma) supplemented with 1 mM Sodium Pyruvate (Sigma), 2 mM L-Glutamine (Sigma), 5% hAB (Sigma) ± 100U/ml interferon gamma (PBL Assay Science) and incubated at 37°C for 16–24 h. Macrophages were then harvested by incubation with Accutase (Sigma) at 37°C to gently detach cells followed by centrifugation at 300 x *g* for 10 min. Cell pellets were resuspended in fresh prewarmed RPMI media (Sigma) supplemented with 1 mM Sodium Pyruvate (Sigma), 2 mM L-Glutamine (Sigma), 5% hAB (Sigma), counted and then diluted to a final concentration of ∼130 cells per microliter to prepare for cell seeding on micropatterns. For targeted gene-specific knockdown, MDM were seeded at 1x10^5^ cells/well and transfected with 60 nM total Silencer Select NHLRC2 (20 nM each of s51529, s51530 and s51531) siRNAs (Ambion) according to the method developed by Fisch et al.,^44^ using the TransIT-X2 Dynamic Delivery System (Mirus Bio) for 48 h followed by the addition of 100u/ml IFN-γ for 16–24 h prior to harvesting and seeding MDMs onto micropatterns. As an siRNA control, 30nM scramble (4390843) siRNA (Ambion) was used.

#### Preparation of microfabricated patterns (micropatterns)

Micropatterns were prepared based on the protocol described in Azioune et al.^22^ Shortly, we coated coverslips with PLL-g-PEG and then made use of a Cyto chromium photomask to imprint the desired size and shape of micropatterns onto coated coverslips. This was then followed by coating of the imprinted micropatterns with Fibronectin.

#### Growing MDMs on micropatterns

Typically, 75,000 MDMs were seeded in a volume of 0.5 mL on coverslips containing fibronectin-coated micropatterns, in a 24-well plate. The cells were left to adhere for 1.5 h, followed by removal of non-adhesive cells by gentle washing with fresh prewarmed RPMI media (Sigma) supplemented with 1 mM Sodium Pyruvate (Sigma), 2 mM L-Glutamine (Sigma), 5% hAB (Sigma), and infection with *Mtb* H37Rv at a multiplicity of infection (MOI) of 10 for 4 h, or *Mtb* Ex52 at a MOI of 10 for 4 or 8 h. This was followed by fixation of cells for 16–24 h with 4% PFA, followed by 1x PBS washes and storage at 4 C for up to one week.

#### Single-cell force cytometry using fluorescently-labeled elastomeric contractible surface (FLECS) technology

MDMs were seeded on Fibronectin-coated elastomeric micropatterns (Forcyte Biotechnologies #F2AX0G03Y, 50 microns, Alexa Fluor 488 - bound Fibrinogen, 24–330 well plate) at a concentration of 75,000 cells per well in prewarmed RPMI media (Sigma) supplemented with 1 mM Sodium Pyruvate (Sigma), 2 mM L-Glutamine (Sigma), 5% hAB (Sigma), and left to adhere and spread for 1.5 h. Unattached cells were then removed and fresh prewarmed RPMI media (Sigma) supplemented with 1 mM Sodium Pyruvate (Sigma), 2 mM L-Glutamine (Sigma), 5% hAB (Sigma), was added, followed by infection with *Mtb* H37Rv at a multiplicity of infection (MOI) of 10 for 4 or 8 h. This was followed by fixation of cells for 16–24 h with 4% PFA, followed by 1x PBS washes and storage at 4 C for up to one week.

#### Staining and immunofluorescence

Fixed samples were washed with 1x PBS, permeabilized for 10 min with 0.1% Triton X-100, washed with 1x PBS and blocked with 3% BSA in 1x PBS. Cells were then incubated with a primary rat monoclonal anti-tubulin antibody (Abcam, #ab6160) at 1:500 concentration for an hour, followed by three washes with 1x PBS and incubation with a secondary antibody conjugated to Alexa Fluor 647 (Abcam, #ab150151) at a concentration of 1:500. Samples were then washed with 1xPBS and incubated with μg/ml DAPI (4′,6-diamidino-2-phenylindole; Life Technologies, #D1306). Coverslips were then mounted in Vectashield (LSBio, #LS-J1032) and taken for imaging. For actin staining, cells were permeabilized for 10 min with 0.1% Triton X-100, washed with 1x PBS and stained with Phalloidin–Atto 565 (SigmaAldrich, #94072). The coverslips were then washed with 1x PBS and incubated with μg/ml DAPI (4′,6-diamidino-2-phenylindole; Life Technologies, #D1306), followed by mounting as described above.

#### Microscopy and image analysis

Mounted samples were imaged on a StellarVision microscope using Synthetic Aperture Optics technology, at a magnification of ×20, on a Zeiss LSM880 airyscan confocal microscope, using a magnification of ×60, and on a Zeiss Axio Observer 7 equipped with a Colibri 7 type RGB-UV LED illumination source, and controlled by ZEN 2.3 (Blue edition) software. For the data collected on the StellarVision, DAPI, Alexa Fluor 488, mCherry and CY5 fluorescence were excited using 385/30 nm, 469/38 nm, 555/30 nm and 631/33 nm, respectively. Raw 16 bit images were saved as.tif files prior to image processing and analysis. For the data collected on the Zeiss Axio Observer, bright-field and fluorescent images were captured using a Zeiss Axiocam 506, using a Plan-Apochromat 100X (1.4NA) Phase Contrast Objective. DAPI, Alexa Fluor 488, mCherry and CY5 fluorescence were excited using 385/30 nm, 469/38 nm, 555/30 nm and 631/33 nm, respectively. Raw 16 bit images were saved as.CZI files prior to image processing and analysis. For the data collected on the Zeiss LSM880 airyscan microscope, DAPI, Alexa Fluor 488, mCherry and CY5 fluorescence were excited using 385/30 nm, 469/38 nm, 555/30 nm and 631/33 nm, respectively. All images were processed and analyzed with FiJi. Image processing included background subtraction and analysis included measurement of distance between MTOC and *Mtb* bacilli, superimposing the quadrant division and quantification of *Mtb* bacilli per quadrant and measurement of fluorescence intensity.

#### Materials

In all our experiments, we stained different cellular elements with different stainings as detailed in the Feature staining table below.

**Table:** 

Feature	Staining
DNA	DAPI - 358⁄461
Fibronectin-coated elastomeric micropatterns (as purchased from Forcyte Biotechnologies #F2AX0G03Y, 50 microns, Alexa Fluor 488 - bound Fibrinogen, 24–330 well plate)	Fibrinogen - Alexa Fluor 488
Tubulin	Alexa Fluor 647

Images were acquired in the TIFF or CZI formats and processed using an automated background noise subtraction algorithm using FiJi.

### Quantification and statistical analysis

Where appropriate, experiments were conducted in technical duplicates or triplicates. Results were expressed as either percentages, absolute values, fold change or copy number (absolute expression per ng cDNA) relative to untreated or non-infected controls for each experiment. Data was analyzed using GraphPad Prism-8 software, Version 8.0, and statistical significance was either analyzed using a Kruskal-Wallis test with Dunn’s multiple comparison, a Mann Whitney test or a Benjamini, Krieger and Yekutieli false discovery rate test. Where appropriate, a 2 tailed t-test was also performed. The statistical details of experiments can be found in the figure legends, figures and Results section.

## Supplemental information

Document S1. Figures S1–S5

Document S2. Article plus supplemental information
