# Supplementary material for: Quantifying spatial dynamics of Mycobacterium tuberculosis infection of human macrophages using microfabricated patterns
Source: Cell Rep Methods. 2023 Nov 13;3(11):100640. doi: 10.1016/j.crmeth.2023.100640 (PMC10694489; doi:10.1016/j.crmeth.2023.100640)
Supplement: Document S1. Figures S1–S5 [file mmc1.pdf]

Cell Reports Methods, Volume *3*

Supplemental information

Quantifying spatial dynamics of *Mycobacterium tuberculosis* infection of human macrophages using microfabricated patterns

Anca F. Savulescu, Nashied Peton, Delia Oosthuizen, Rudranil Hazra, Robert P. Rousseau, Musa M. Mhlanga, and Anna K. Coussens

SUPPLEMENTARY FIGURES

Figure S1: Optimization of the protocol on round versus crossbow shaped micropatterns, related to Figure 1

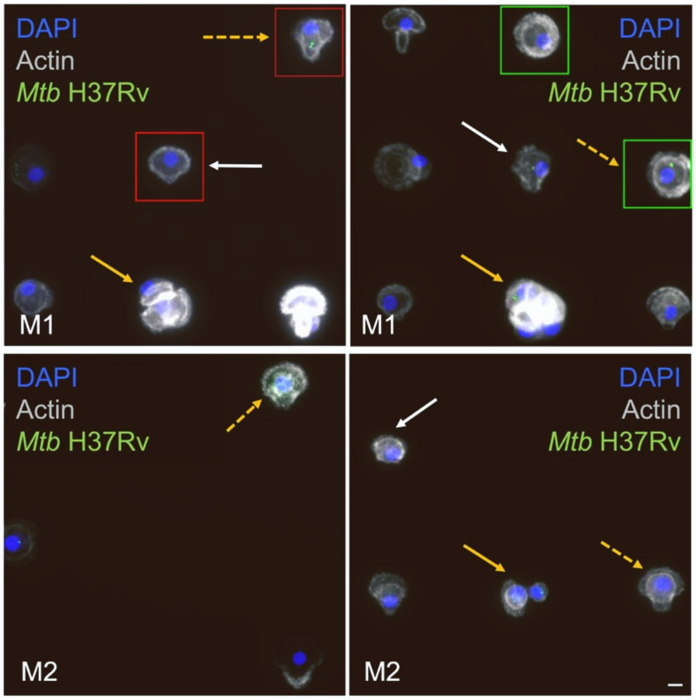

Figure S1. Optimization of the protocol on round versus crossbow shaped micropatterns. M1 and M2 MDM cells were seeded and grown on medium sized crossbow (examples in red squares) or round shaped (examples in green squares) micropatterns and infected with H37Rv, as detailed in Figure 1A. Orange dotted arrows indicate cells that spread properly on micropatterns, orange arrows indicate multiple cells on one micropattern and white arrows indicate cells that did not spread properly on micropatterns. The DNA is stained with DAPI (blue), H37Rv is tagged with GFP (green) and actin is stained with phalloidin (gray). Scale bar 10 µm.

Figure S2: Live *Mtb* increases phagocytosis compared to heat-killed *Mtb*, related to Figure 2

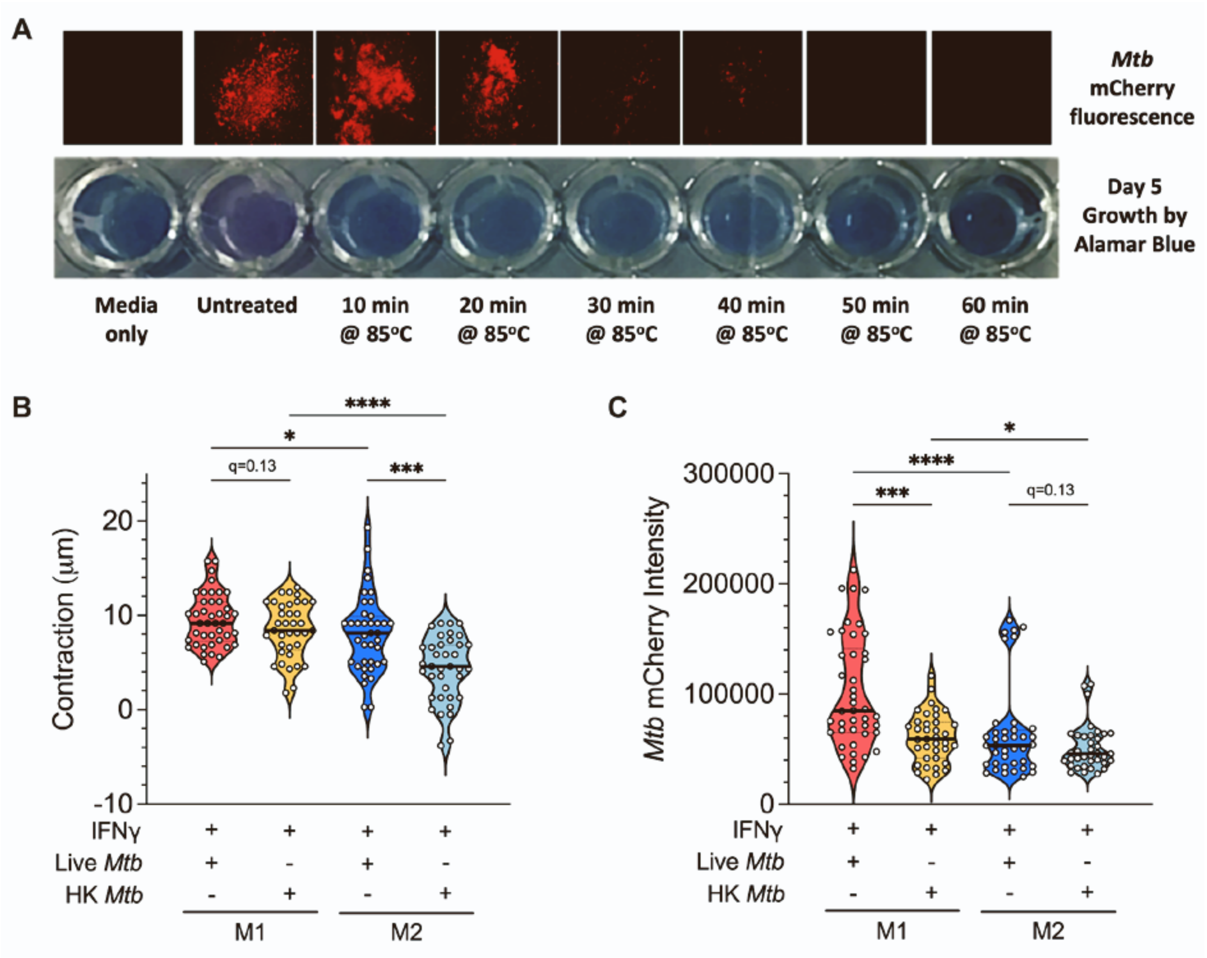

Figure S2. Live *Mtb* increases phagocytosis compared to heat-killed *Mtb*. **A**. *Mtb* was heat-killed at 85°C for increasing 10 min intervals from 10 to 60 min. *Mtb* mCherry fluorescence fully dissipated after 50 min. An aliquot of each was innoculated in 7H9/ADC broth and after 5 days incubation at 37°C, viability was measured using alamarBlue assay which turns pink in the presence of viable cells and remains blue in absence of growth, showing *Mtb* was killed after 10 min at 85°C. **B**. Contraction (measured in µm) of micropatterns for M1 and M2 MDMs treated with IFN-γ and infected for 4 hours with live or 10 min heat-killed (HK) *Mtb*. **C**. Mean fluorescence intensity (MFI) of live and HK *Mtb* in M1 and M2 MDM cells, after 4 hours phagocytosis. n=2 donors. Violin plot line, median, dotted lines, IQR; analyzed by Kruskall-Wallis test with Benjamini, Krieger and Yekutieli false discovery rate (q-value); *, q<0.05; ***, q<0.001; ****, q<0.0001.

Figure S3: Absolute number of MDMs adhered and correctly spread on crossbow shaped micropatterns at different time points post infection with H37Rv, related to Figure 3

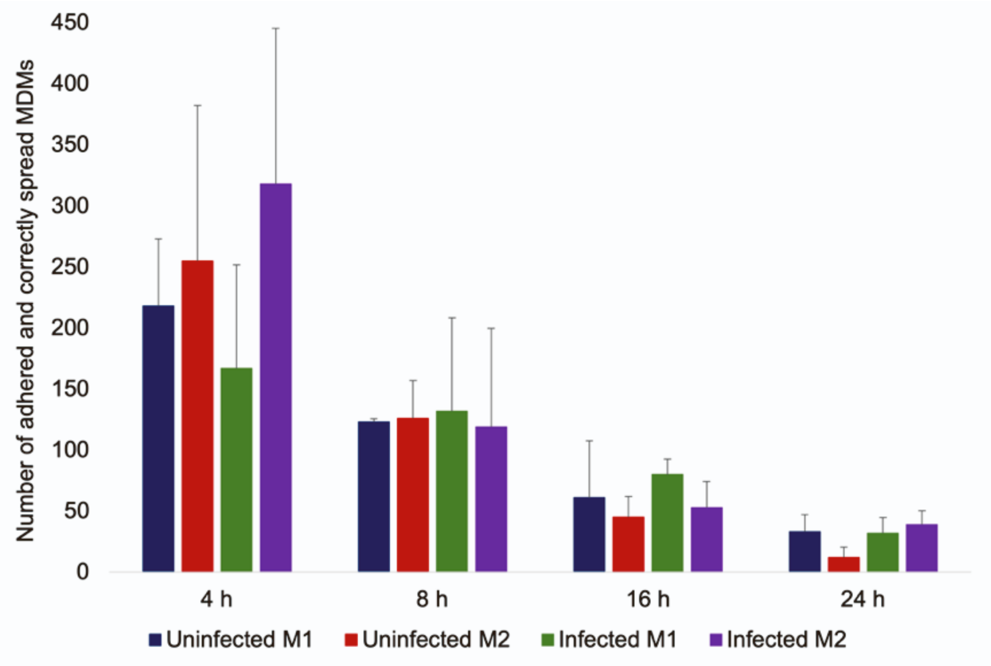

Figure S3. Absolute number of MDMs adhered and correctly spread on crossbow shaped micropatterns at different time points post infection with H37Rv. n=3 donors, mean ± SD. P=0.832 for uninfected M1s vs M2s at 4 h; P=0.476 for infected M1s and M2s at 4 h; P=0.725 for uninfected versus infected M1s at 4 h; P=0.772 for uninfected versus infected M2s at 4 h. Analyzed by 2 tailed T-test.

Figure S4: Typical H37Rv infected M1 and M2 MDMs on round shaped micropatterns, related to Figure 3

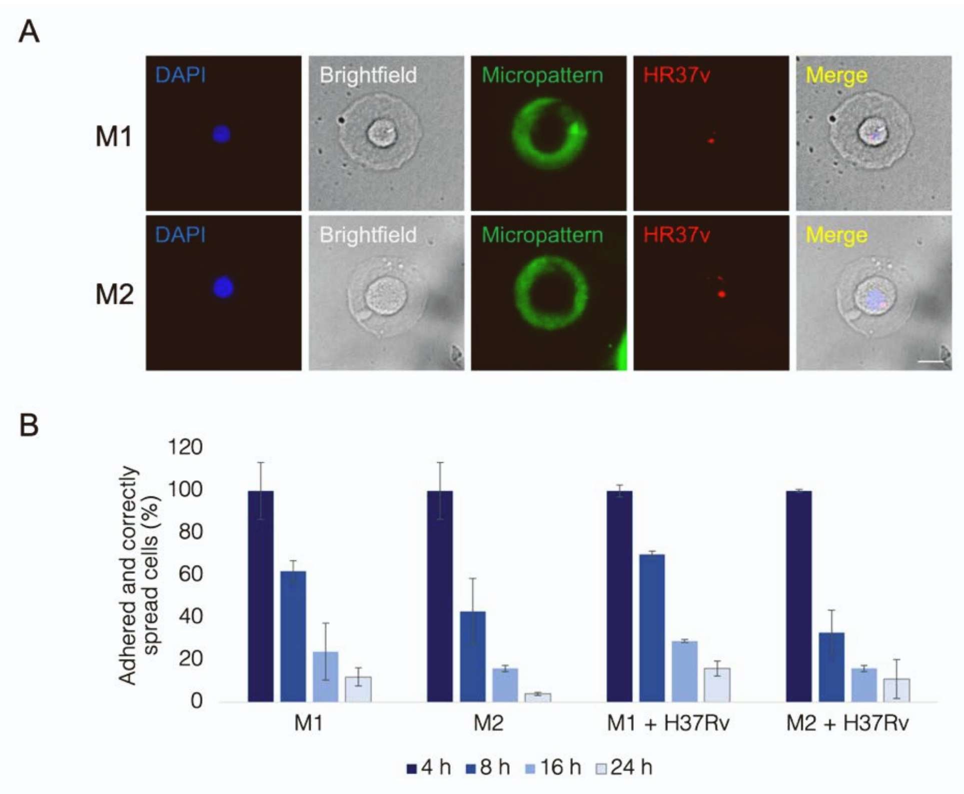

Figure S4. A. Typical H37Rv infected M1 and M2 MDMs on round shaped micropatterns. The DNA is labeled with DAPI (blue), the cell can be seen in brightfield, the micropatterns are labeled with AlexaFluor-488 (green), *Mtb* H37Rv expresses mCherry (red) and a merged image (combining all fields other than the green micropattern, for clarity) is shown on the right. Scale bar 10 µm. **B.** Percentages of appropriately adhered and spread, uninfected and H37Rv infected M1 and M2 MDMs on round shaped micropatterns at 4, 8, 16 and 24 hours post infection. The number of cells that are adhered and spread in an appropriate manner per condition at 4 h is considered as the 100 %, with the following time points calculated as a percentage of the 4 hours. n=3 donors, mean ± SD.

Figure S5: Example images of H37Rv infection in MDM, related to Figure 4

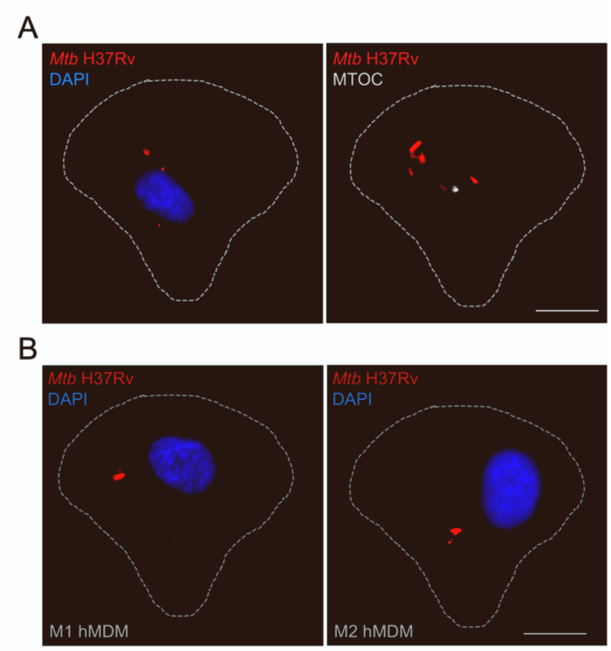

Figure S5. Example images of H37Rv infection in MDM. **A**. M2 MDM cells infected with H37Rv where the nucleus (left image) and MTOC (right image) are positioned correctly. The DNA is stained with DAPI (blue), H37Rv is tagged with mCherry (red) and the MTOC is marked in gray (microtubules and MTOC were stained with anti Tubulin and the background was removed to emphasize the MTOC), the contour of the cell is shown in dotted line. Scale bar 10 µm. **B**. Example images of H37Rv infected MDMs (M1 on the left and M2 on the right) in which the nucleus is not positioned correctly. The DNA is stained with DAPI (blue), H37Rv is tagged with mCherry (red), scale bar 10 µm.
